# Measured Resection as Gap Balance Method in Mobile‐Bearing Medial Unicompartmental Knee Arthroplasty: A Randomized Controlled Trial

**DOI:** 10.1111/os.14346

**Published:** 2025-01-05

**Authors:** Qian Liu, Jianhua Ren, Wenhui Zhang, Tangzhao Liang, Zhe Wang, Siwei Xie, Yuhang Li, Jianfeng Hou, Kun Wang, Ronghan He

**Affiliations:** ^1^ Department of Joint and Trauma Surgery The Third Affiliated Hospital of Sun Yat‐Sen University Guangzhou China; ^2^ Department of Environmental Health and Engineering Johns Hopkins Bloomberg School of Public Health Baltimore Maryland USA

**Keywords:** clinical outcome, gap balance, meniscal bearing thickness, mobile‐bearing unicompartmental knee arthroplasty, resected bone pieces

## Abstract

**Objective:**

Gap balancing is a vital process during mobile‐bearing unicompartmental knee arthroplasty (MB‐UKA). However, this process commonly depends on the surgeon's experience and lacks specific unified standards. This study aimed to propose and evaluate a novel “measured resection” method for gap balance in MB‐UKA.

**Methods:**

This prospective study included 49 consecutive patients (52 knees) who underwent MB‐UKA from February 1, 2023, to September 1, 2023. Gap balance was achieved by the traditional “two‐finger” method (Group 1, 26 knees) or the measured resection method (Group 2, 26 knees). The novel “measured resection” method was performed by measuring the thickness of the resected posterior femoral condyle and resected medial posterior tibial plateau to assess proper meniscal bearing thickness. Data were collected at baseline and the 6‐month follow‐up. Prosthetic angles, range of motion (ROM), visual analog scale (VAS) score, Oxford knee score (OKS), and Global Perceived Scale (GPE) were used to evaluate clinical outcomes. Independent samples *t*‐test and Mann–Whitney *U* test were used to compare the differences.

**Results:**

There were significant improvements in all measured outcomes at the 6‐month follow‐up from baseline in both groups (*p* < 0.01). Patients using measured resection method showed better ROM (130° vs. 120°, *p* = 0.007), VAS score (1 vs. 2, *p* = 0.013), and OKS scores (39.9 vs. 38.1, *p* = 0.013) at 6‐month follow‐up than the traditional “two‐finger” method group. The prosthetic angles, ROM improvement, and GPE showed no significant difference between the groups (*p* > 0.05).

**Conclusions:**

The measured resection method is a reliable method for assisting surgeons in choosing the ideal meniscal bearing thickness in MB‐UKA to achieve proper gap balance and gain better clinical outcomes.

**Trial Registration:**

ClinicalTrials.gov (NCT03815448)

## Graphical abstract

By applying the measured resection method in MB‐UKA for gap balancing, the surgeons can select optimal meniscal bearing thickness and gain better clinical outcomes than the traditional method.

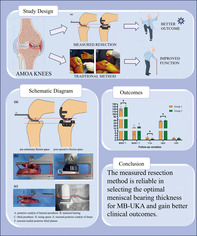

**Abbreviations**
ACL: anterior cruciate ligament
AMOA: anterior medial knee osteoarthritis
GPE: Global Perceived Scale
MB‐UKA: mobile‐bearing UKA
MCL: medial collateral ligament
MRI: magnetic resonance imaging
OKS: Oxford knee score
ROM: range of motion
TKA: total knee arthroplasty
UKA: unicompartmental knee arthroplasty
VAS: visual analog scale

## 1 Introduction

Unicompartmental knee arthroplasty (UKA) is an effective and commonly used surgical technique for patients with anterior medial knee osteoarthritis (AMOA) [1, 2]. Compared to total knee arthroplasty (TKA), UKA has the advantages of less surgical trauma and intraoperative bleeding, shorter surgical time, faster postoperative recovery, and better kinematics and functional scores [3, 4]. Thus, the Oxford mobile‐bearing UKA (MB‐UKA) system is slowly gaining global popularity.

Balancing extension and flexion gaps in MB‐UKA is a vital step, as it directly affects the effectiveness and outcome of the surgery. A tight gap will result in prosthetic aseptic loosening, accelerated bearing wear, and aggravation of lateral compartment osteoarthritis [5, 6]. A loose gap increases the risk of postoperative pain, knee instability, and bearing dislocation [7, 8]. Therefore, a precise gap balance is crucial. The current surgical technique design and surgical instruments enable surgeons to perform accurate bone resection, but lack specific and quantified standard when surgeons aim to restore soft tissue tension in the medial compartment. Soft tissue release is strictly prohibited in MB‐UKA to avoid medial collateral ligament (MCL) damage, choosing the appropriate meniscal bearing is the key to proper gap balance [9]. According to the Oxford Partial Knee manual [10], surgeons achieve natural ligament tension when they hold the feeler gauge with two fingers and can easily slide it in and out without tilting, which is also known as “two‐finger” method to some surgeons. This traditional method highly relies on the surgeon's hand feel and varies among them, causing fluctuating outcomes [11]. This poses difficulties for the standardized implementation of MB‐UKA and is not conducive for novice surgeons to carry out this surgery, which is one of the reasons for the steep learning curve of UKA and the occurrence of complications. Furthermore, femoral resection in UKA is a millimeter‐level process, relying on a rough tactile sense to guide such a precise process obviously poses a hidden risk of error.

Researchers have carried out multiple studies on how to quantify and standardize the process of soft tissue balancing during MB‐UKA surgery. Some researchers have focused on designing tensors [12] or pressure sensors [13] to measure the tension within the joint space, aiming to achieve pressure balance between the flexion and extension gaps during surgery. The tensors are capable of exerting a consistent force onto the joint space, thereby restoring tension to the MCL. By measuring the distracted joint space distance, surgeons can evaluate the balance between extension and flexion. However, the distracting force is closely related to the relaxation state of the MCL, and there is currently no consensus on the most appropriate level of force [14, 15]. Pressure sensors can measure interprosthetic pressure through electrodes and pressure sensitive materials, converting different pressures into resistance values and finally output specific pressure values. This enables surgeons to conduct in vitro explorations [16] or apply it intraoperatively to analyze the relationship between joint pressure and postoperative indicators [17]. However, factors including the design of sensors and surgical positioning can significantly impact the flexion and extension pressures during MB‐UKA [18]. Meanwhile, robotic‐assisted MB‐UKA is a more individualized, high‐precision yet more expensive surgical method. Using robotic assistance can enhance accuracy of component positioning and alignment and reduce the occurrence of postoperative complications [19]. Jenny et al. [20] discovered more details about soft tissue balancing through the use of intraoperative navigation systems. The robotic‐assisted surgery can minimize the impact of joint laxity on manual sensation and exclude the errors associated with the traditional two‐finger method [21]. Conducting MB‐UKA with robotic assistance has also been shown to achieve favorable soft tissue balancing [22]. These devices can provide objective and quantifiable data that can assist surgeons in assessing and adjusting the soft tissue balancing, each of these methods has its own advantages and disadvantages. Currently, there is no consensus on how to accurately quantify the process of soft tissue balancing, the variations in tactile sensation among different surgeons still exert a considerable influence on the efficacy of soft tissue balancing and clinical outcomes.

Therefore, it is necessary to excogitate a reliable method to help correct the traditional method for gap balancing. This method should be easy to operate and master without significantly increasing the duration and risks of the surgery. Ideally, it should not rely on additional equipment or instruments to achieve the process, thereby enhancing its universal applicability. If a reasonable and precise method for soft tissue balancing can be successfully designed, surgeons can perform MB‐UKA more effectively, and it is anticipated that postoperative pain and the occurrence of prosthetic complications can be reduced, thereby enhancing patient satisfaction and survival rates. In this study, we aim to design a simple, easy‐to‐implement, and instrument‐free method for soft tissue balancing, with the expectation that patients undergoing MB‐UKA using this method will experience less postoperative pain and achieve higher functional outcomes. If this method proves to be effective, it has the potential to be widely applied and assist experienced doctors in performing soft tissue balancing more precisely, while also guiding novice doctors in learning how to conduct MB‐UKA surgery more smoothly. Based on the anatomical and disease characteristics of AMOA patients, as well as the surgical and prosthetic design principles of MB‐UKA, we designed a novel “measured resection” method to assess meniscal bearing thickness for appropriate gap balance. The proposes of the study were to (i) preliminarily evaluate the feasibility and reliability of this “measured resection” method, (ii) compare the differences in various postoperative follow‐up indicators between the “measured resection” method and the traditional “two‐finger” method, and (iii) explore the short‐comings and areas for improvement in the “measured resection” method.

## 2 Materials and Methods

### 2.1 Principles of Measured Resection Method

**FIGURE 1:**
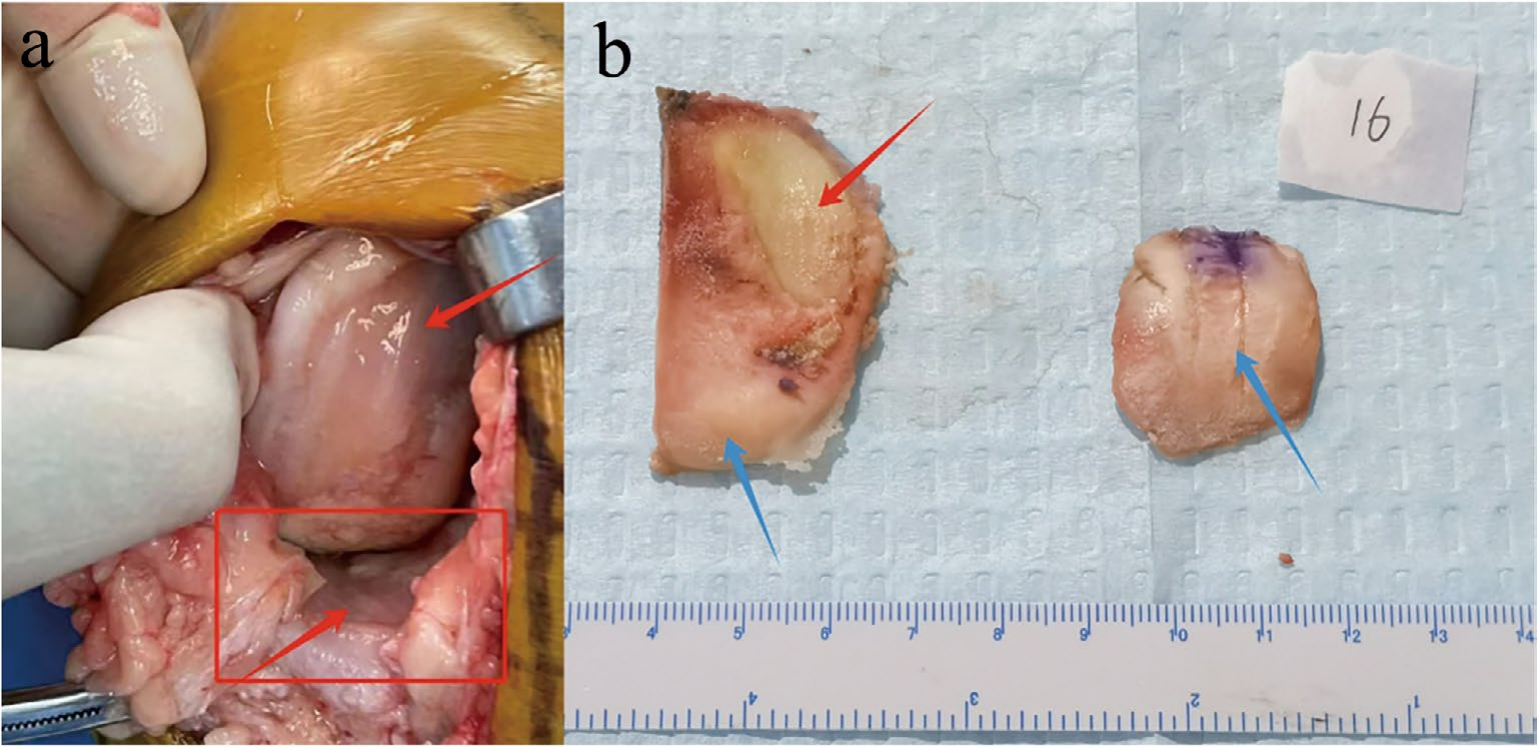
(a) Intraoperative observations of anterior medial knee osteoarthritis. (b) Resected bone pieces of anterior medial knee osteoarthritis patient. The red arrows referred to cartilage damage and bone defect on anterior medial tibial plateau and distal femur, and the blue arrows referred to normal cartilage on the medial posterior tibial plateau and posterior femoral condyle.

Measured resection method determines meniscal bearing thickness based on the thickness of intraoperative resected bone pieces (posterior femoral condyle and medial posterior tibial plateau) and thickness of surgical instruments and prostheses (femoral sizing spoon, femoral and tibial prostheses). In patients with AMOA, cartilage injury is mainly limited to the anterior medial compartment, while the posterior articular cartilage remains relatively unscathed (Figure 1).

**FIGURE 2:**
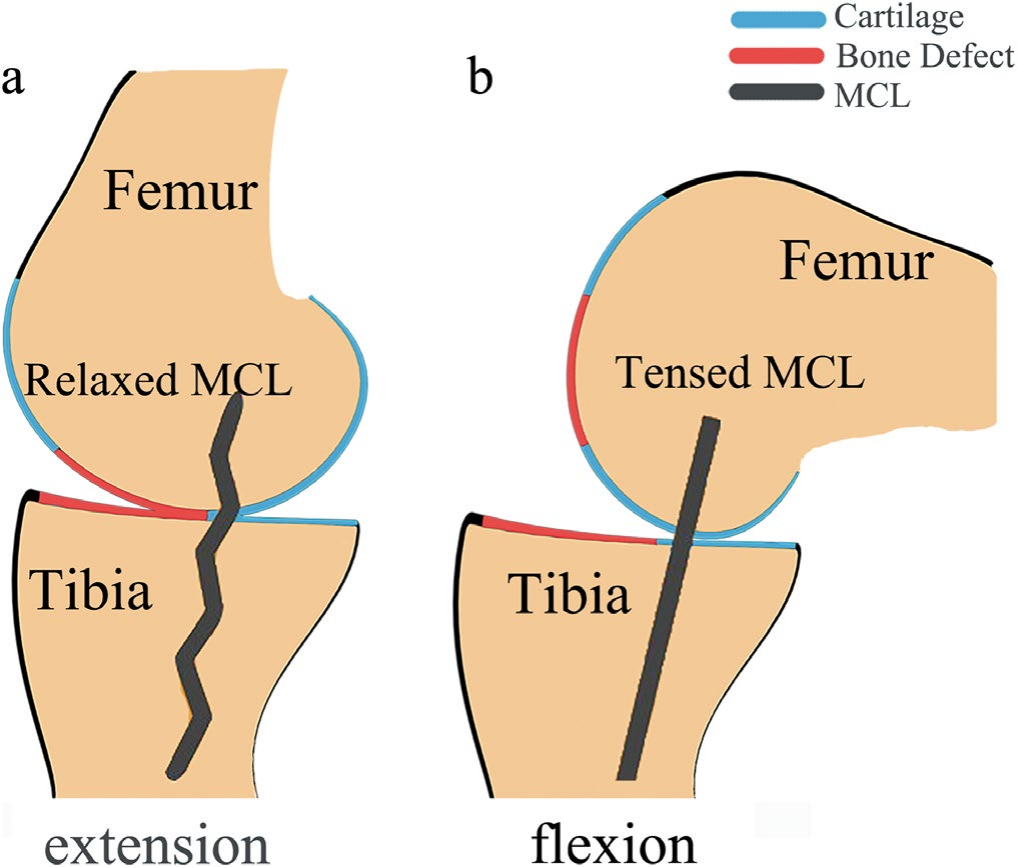
Demonstration of anterior medial knee osteoarthritis. (a) Knee extension. MCL relaxes in knee extension due to bone defect in the anterior medial compartment; (b) knee flexion. MCL tenses in knee flexion because cartilage on the medial posterior tibial plateau and femoral condyle remain intact.

When the knee is extended (Figure 2), anterior medial compartment cartilage defect will cause varus deformity with relaxed MCL [23]. According to the magnetic resonance imaging (MRI) scan results of Nakagawa et al. [24] on the normal knee joint flexion and extension process, the contact point between the medial femoral condyle and the medial tibial plateau is located in the middle and rear parts of the medial tibial plateau during knee flexion from 90° to 133°. At this time, the intact joint cartilage of the medial tibial plateau and the medial femoral condyle are in contact with each other, and the MCL is tense. The overall knee joint relationship is close to a normal flexed knee. Although MCL flexion contracture might be remained to some degree, but that can be corrected according to the MB‐UKA indications [25], by selecting an appropriate femoral sizing spoon during surgery. We named the flexion gap composed of the posterior femoral condyle, the femoral sizing spoon, and the posterior part of the medial tibial plateau as the “preosteotomy flexion gap”. After proper gap balancing and prosthesis insertion, the flexion gap composed of posterior femoral prosthesis, sniscal bearing and tibial prosthesis is named “postoperative flexion gap”.

**FIGURE 3:**
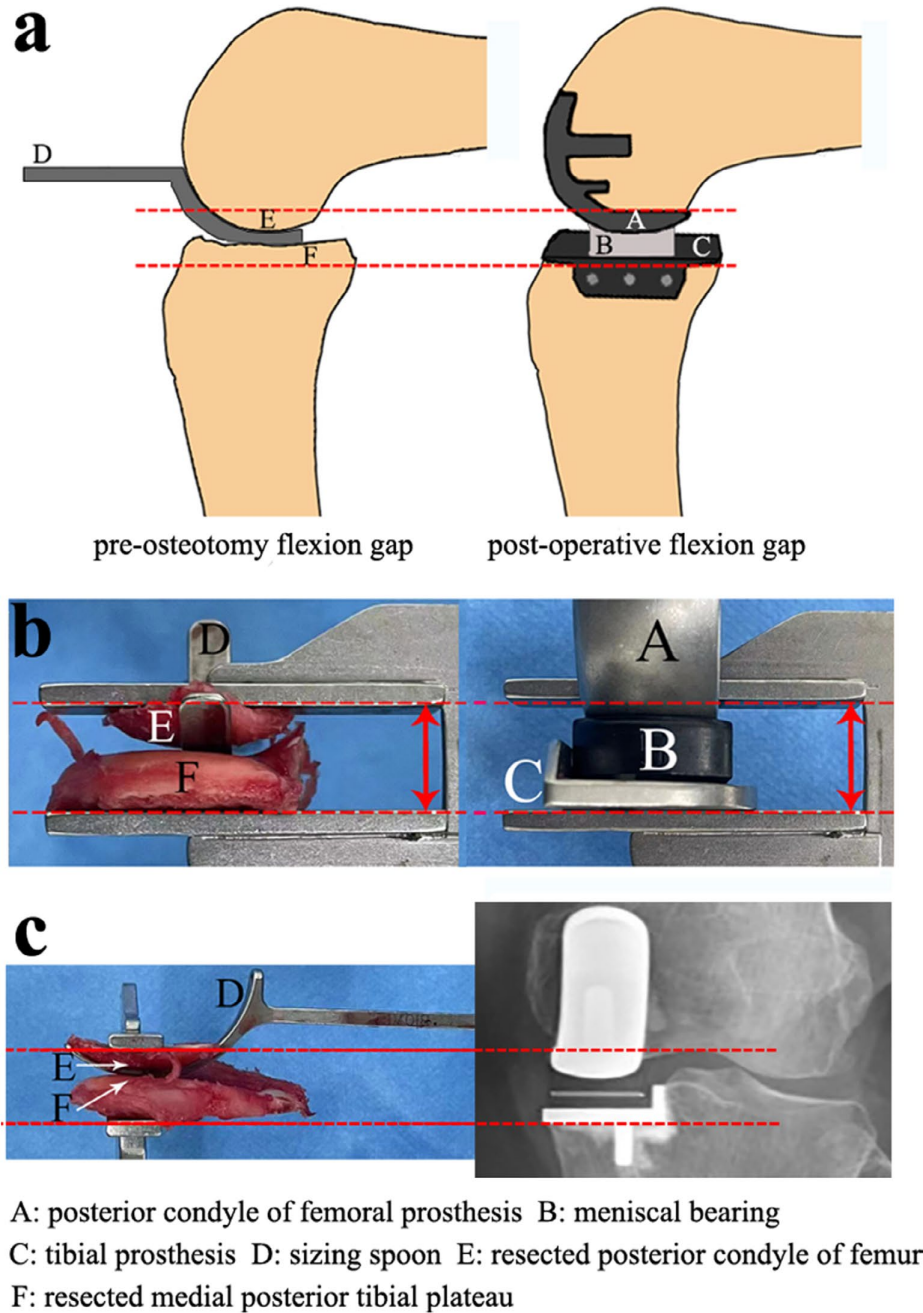
(a) Theoretical schematic diagram of measured resection method. The postoperative flexion gap should be in accord with the preosteotomy flexion gap. (b) Anterior–posterior view of resected bone + sizing spoon versus joint prosthesis + meniscal bearing. (c) Lateral view of resected bone + sizing spoon versus joint prosthesis + meniscal bearing. The thickness of A + B + C should be equal to the thickness of D + E + F to achieve a satisfying postoperative function.

Since the preosteotomy flexion gap corrects flexion contracture and is close to an individual's normal knee joint, the knee joint in this state should be able to ensure relatively good functionality. We should expect good surgical efficacy if the postoperative flexion gap is equal to the preosteotomy flexion gap (Equation (1)). Because the preosteotomy flexion gap is composed of the femoral sizing spoon and the bone to be resected, we can determine the appropriate meniscal bearing thickness based on the thickness of the resected bone fragments. The thickness of the resected femoral and tibial bone pieces can be measured intraoperatively, and the thicknesses of used femoral sizing spoon and prostheses are accessible via the manufacturer's manual. The ideal meniscal bearing thickness can be calculated by our measured resection method (Equation (2)). Theoretical and intraoperative pictures are reported in Figure 3.

(1) $$\begin{array}{c} \text{Resected femoral condyle}+\text{Resected medial posterior tibial plateau}+\text{Sizing spoon}=\\ \text{Posterior femoral condyle}+\text{Tibial plateau}+\text{Meniscal bearing prosthesis} \end{array}$$

(2) $$\begin{array}{c} \text{Meniscal bearing prosthesis}=\\ \left(\text{Resected femoral condyle}+\text{Resected medial posterior tibial plateau}+\text{Sizing spoon}\right)\\ -\left(\text{Posterior femoral condyle}+\text{Tibial plateau prosthesis}\right) \end{array}$$

### 2.2 Patient Recruitment

**FIGURE 4:**
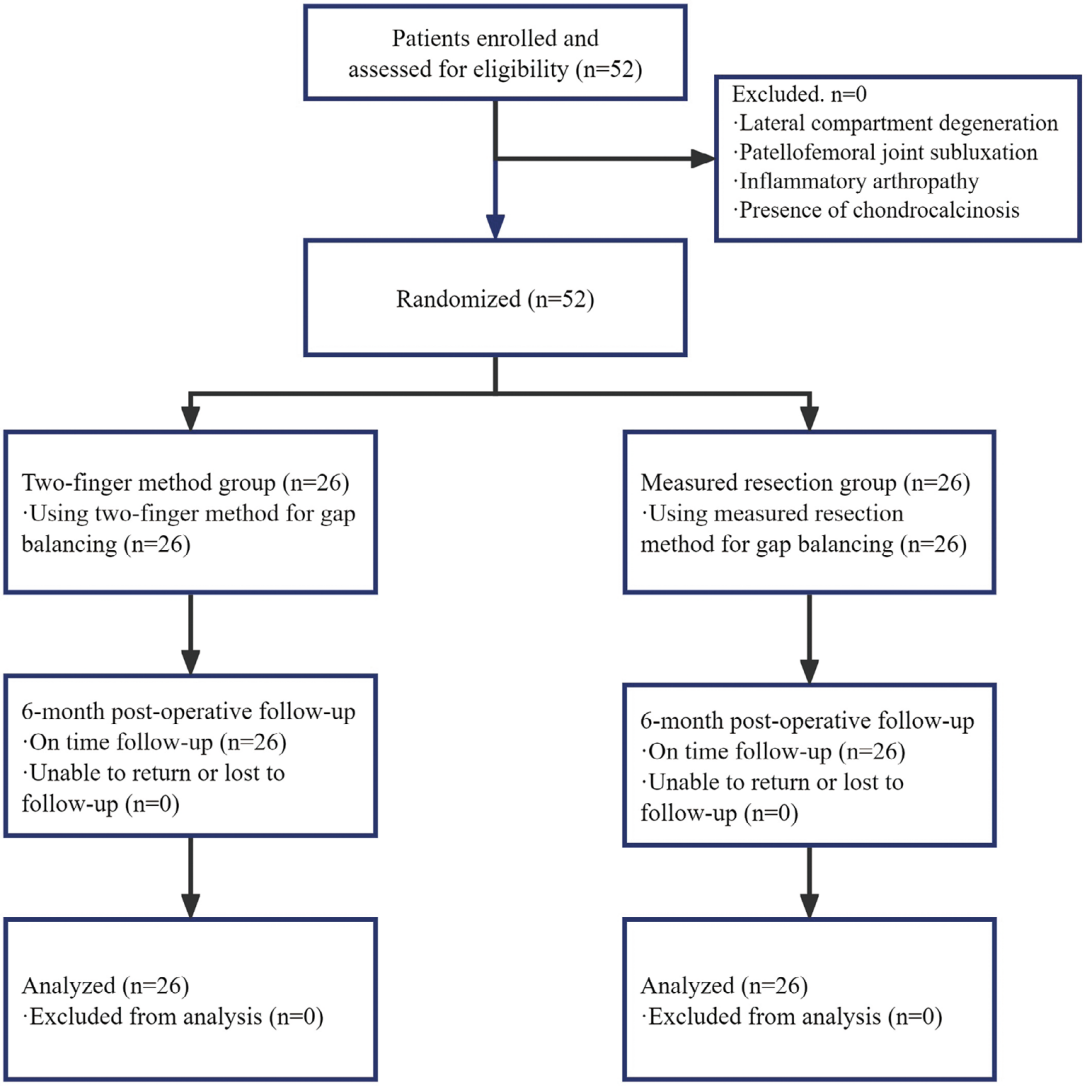
Study flow chart.

This was a prospective, randomized, parallel, single‐center study. There were 52 knees (49 patients) operated with MB‐UKA from February 1, 2023, to September 1, 2023, in our department (Figure 4). The inclusion criteria for surgery were AMOA, age > 50 years old, BMI < 32 kg/m^2^ and Kellgren & Lawrence grade 3 or 4 osteoarthritis in the medial knee compartment [26]. The indications for MB‐UKA are fixed flexion deformity of the knee < 15°, active ROM > 90°, varus deformity < 15° and can be manually corrected, functional MCL confirmed by varus and valgus stress radiograph of the knee, intact anterior cruciate ligament confirmed by MRI. The exclusion criteria were patients combined with degeneration of knee lateral compartment, subluxation of the patellofemoral joint, inflammatory arthropathy, and presence of chondrocalcinosis. The methods and procedures for our study were approved by the Ethics Review Committee of the Third Affiliated Hospital of Sun Yae‐Sen University, study number II 2024–011‐01. All participants included signed informed consent.

In this study, we randomly assigned 52 knees into two groups: the two‐finger method group (*n* = 26), in which the traditional two‐finger method was used for gap balance, and the measured resection group (*n* = 26), in which the measured resection method was used for gap balance. Randomization was performed using a computer‐generated random number sequence to allocate patients into either the two‐finger method group or the measured resection group in a 1:1 ratio. The randomization sequence was generated by a third party who was not involved in the recruitment or allocation of patients to minimize the risk of selection bias. The patients were blinded to their group assignment, but the surgeons were not blinded due to the nature of the study. Besides, physicians conducting follow‐up visits, data collection, and data analysis were blinded to the patient grouping to minimize bias. Basic information of the patients is reported in Table 1.

**TABLE 1:** Patient demographics.

Baseline data	“Two‐finger” method (*n* = 26)	Measured resection (*n* = 26)	*p*
Age (years)	66.8 ± 8.7	68.5 ± 6.9	0.439
Sex (men: women)	23%: 77%	19%: 81%	0.734
Body mass index (kg/m^2^)	25.52 ± 2.74	26.92 ± 3.97	0.146
Operation side (left: right)	50%: 50%	46%: 54%	/
Range of motion (°)	105 (100, 120)	110 (100, 120)	0.452
Visual analog scale (0–10)	6.00 (5.00, 6.00)	6.00 (5.00, 6.25)	0.827
Oxford knee score (0–48)	29.7 ± 2.0	29.1 ± 2.5	0.362

### 2.3 Sample Size

The sample size of the study was based on the mean preoperation and postoperation OKS score documented in pre‐experimental questionnaire survey, which was reported to be 28.8 and 38.8, respectively, and the sample size ratio was 1:1. With an *α* level of 0.05 and a power of 90%, the sample size was at least 52 patients, calculated by PASS 2021 (Power Analysis and Sample Size, NCSS LLC, USA) [27].

### 2.4 MB‐UKA Surgical Procedure

All surgeries in both groups were performed by one experienced senior doctor, who has performed more than 50 MB‐UKAs per year for more than 20 years. All surgical procedures in both groups were completed under combined spinal‐epidural anesthesia and using the third generation Oxford MB‐UKA prosthesis and meniscal bearing (Zimmer, Biomet, Warsaw, IN, USA). The surgical position, tourniquet, and approach were consistent with the manufacturer's manual [10]. The intact anterior cruciate ligament and lateral compartment cartilage were examined, and there was no shift from MB‐UKA to TKA.

After inserting in tibial and femoral trial implants, the surgeon achieved gap balance at 20° and 110° of knee flexion based on the “two‐finger” method in Group 1, by which they could hold the chosen spacer blocker with two fingers and easily insert and remove it without resistance. Group 2 received measured resection as gap balance method. The subsequent surgical procedures for both groups of patients were consistent and were performed in accordance with the manufacturer's manual [10].

### 2.5 Measured Resection

Thicknesses of the resected posterior femoral condyle, resected medial posterior tibial plateau, and sizing spoon were measured and recorded. Measurements were done by one doctor using one electronic vernier caliper (Yantai Greenery Tools Co. Ltd., Shandong, China), repeatedly measured 3 times for each specimen, took average if the results demonstrate no significant dispersion, and accuracy was taken to 1 decimal place. The measurement points for the femoral specimen are the osteotomy plane and the thickest point of the specimen. For the tibial specimen, the measurement points include the osteotomy plane and the contact area with the femur when the knee is flexed at 90°–133° [24]. It should be noted that if the calculated meniscal bearing thickness did not equal to an integral number, a thinner bearing was chosen without increasing the risk of dislocation. For example, if the calculated meniscal bearing thickness was greater than 4 mm and less than 4.8 mm, a 4‐mm bearing was chosen. A further gap balance was done by two‐finger method to access and record ideal meniscal bearing thickness based on the traditional method.

### 2.6 Postoperation Evaluations and Follow‐Up

**FIGURE 5:**
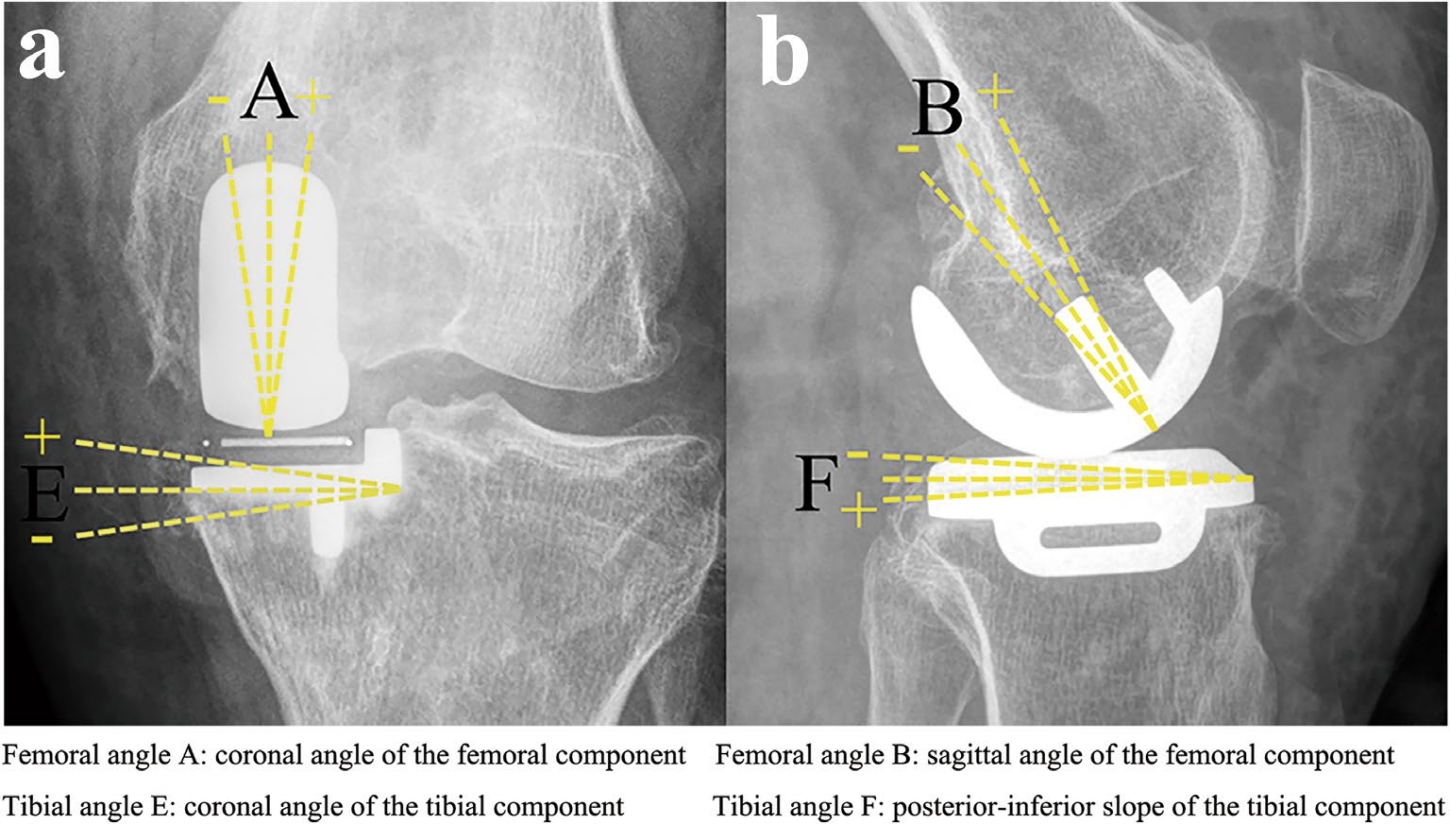
Radiological assessment of the component position. (a) Anterior–posterior view of femoral angle A and tibial angle E. (b) Lateral view of femoral angle B and tibial angle F.

Both groups of patients received the same routine postoperative nursing and medication. Standardized anterior–posterior and lateral supine knee X‐rays were taken and analyzed in all patients on postoperative day 1. The femoral component angle A (angle between the axis of the component and axis of the lower limb) and the tibial component angle E (angle between the axis of the tibial component and horizontal line) were recorded on the anterior–posterior X‐ray. Additionally, the femoral component angle B (angle between the peg axis of the femoral component and axis of the femoral stem) and the tibial component angle F (posterior‐inferior slope of the tibial component) were recorded on the lateral X‐ray (Figure 5). The 10‐point pain visual analog scale (VAS), range of motion (ROM), and Chinese version Oxford knee score (OKS) were collected preoperative (baseline) and at the 6‐month follow‐up [28]. The Global Perceived Scale (GPE) was also collected at the 6‐month follow‐up.

### 2.7 Statistical Analysis

All statistical analyses were performed using SPSS version 26 software (IBM Corp., Armonk, NY, USA). Normality of data distribution was assessed using the Shapiro–Wilk test, and data satisfying normal distribution are represented by mean ± SD, while data with nonnormal distribution are represented by median (Q1, Q3). After performing the Shapiro–Wilk test, the variables that satisfied the normal distribution were age (*p* = 0.079), BMI (*p* > 0.2), prosthetic angles (*p* > 0.05), and OKS score (*p* > 0.2). The variables that did not satisfy the normal distribution were ROM, ROM improvement, GPE, and VAS score (*p* < 0.01). Independent samples *t*‐test was used for statistical analysis following normal distribution. The chi‐square test was used to examine the baseline gender ratio. The Mann–Whitney *U* test was used to compare the ROM, ROM improvement, VAS score, and GPE score. The bar charts and schematic diagrams were respectively created using GraphPad Prism 10.1.2 (San Diego, California, USA) and Photoshop 2023 (Adobe Inc., San Jose, CA, USA), all of the figures were owned by the authors.

## 3 Results

### 3.1 Data of Measured Resection

Detailed data of the measured resection group were reported in Table 2. Meniscal bearing thickness was calculated by Equation (2). There were 16 patients with definite gap balance achieved by the “two‐finger” method, and the results were in accord with measured resection method. The meniscal bearing thickness for other 10 patients were chosen based on the measured resection method.

**TABLE 2:** Data of measured resection group.

Number	Resected tibial (mm)	Resected femoral condyle (mm)	Sizing spoon (mm)	Femoral prosthesis	Tibial prosthesis	Bearing thickness by measured resection (mm)	Bearing thickness by two‐finger method(mm)	Final choice of bearing (mm)
1	6.1	5.1	1	XS	A	3.7	3–4	3
2	5.4	5.3	1	XS	AA	3.2	3	3
3	6.4	4.9	2	XS	A	4.8	4–5	5
4	7.1	4.6	1	S	B	3.7	3–4	3
5	6.9	5.9	1	S	A	4.8	5	5
6	5.8	5.4	1	XS	A	3.7	4	4
7	5.3	5.5	2	S	B	3.8	4	4
8	6.9	5.8	1	S	A	4.7	4–5	4
9	6.7	4.7	1	XS	A	3.9	4	4
10	7.3	5.2	1	XS	AA	5	5	5
11	5.1	5.5	1	XS	A	3.1	3	3
12	7.5	4.5	1	XS	A	4.5	4	4
13	8.6	4.2	1	S	A	4.8	4–5	5
14	6	5.3	1	XS	B	3.8	4	4
15	5.5	6.1	1	XS	A	4.1	4	4
16	4.4	6	2	S	C	3.4	3–4	3
17	5.3	6.3	2	S	B	4.6	4–5	4
18	4.8	7.5	1	S	A	4.3	4	4
19	5.5	5.7	1	XS	A	3.7	3–4	3
20	5.4	6	1	M	D	3.4	3–4	3
21	4.1	5.5	2	XS	A	3.1	3	3
22	5.7	6	2	S	A	4.7	4–5	4
23	6.2	4.6	1	XS	A	3.3	3	3
24	6.2	3.7	3	S	AA	3.9	4	4
25	6.5	5.9	1	S	C	4.4	4	4
26	7.1	4.8	3	S	B	5.9	6	6

### 3.2 Postoperative Variables of the Patients

**FIGURE 6:**
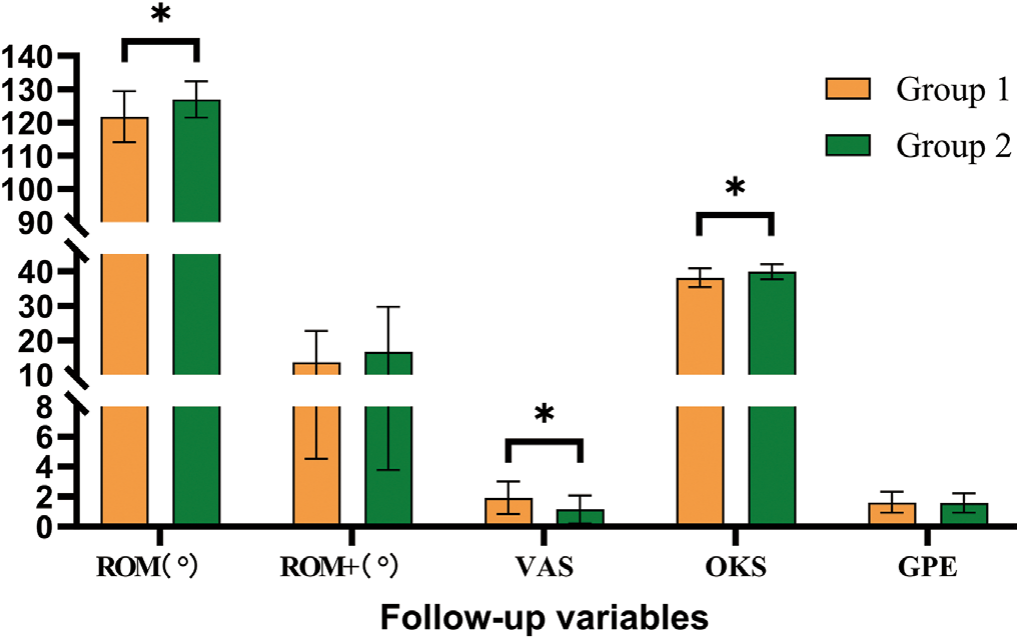
Patient's clinical outcomes at 6‐month follow‐up. *: statistical significance; ROM: range of motion; ROM+: range of motion improvement; VAS: visual analog scale scores; OKS: Oxford knee score.

The clinical outcomes we concerned about were mainly related to pain relief and knee joint function improvement of patients, so we selected ROM, VAS, OKS score, and GPE for recording. The prosthetic angles were also measured to evaluate whether measured resection will affect implant position, which may cause complications or biomechanics changes. Significant improvements were found in postoperative ROM, VAS, and OKS scores compared with preoperative data (*p* ﹤ 0.01) in both groups, reported in Table 3. Besides, measured resection group showed better ROM, VAS, and OKS scores at 6‐month follow‐up than the “two‐finger” method group (*p* ﹤ 0.05). The prosthetic angles, ROM improvement, and GPE showed no statistical difference between the groups (*p* ﹥ 0.05), which means measured resection did not affect implant angles compared with the traditional method, reported in Table 4 and Figure 6. No severe postoperative complications were found.

**TABLE 3:** Preoperative and postoperative variables comparisons.

Variables	“Two‐finger” method group (*n* = 26)			Measured resection group (*n* = 26)		
	Baseline	6‐month	*p*	Baseline	6‐month	*p*
Range of motion (°)	105 (100,120)	120 (115,130)	< 0.001	110 (100,120)	130 (120,130)	< 0.001
Visual analog scale (0–10)	6.00 (5.00, 6.00)	2.00 (1.00, 3.00)	< 0.001	6.00 (5.00, 6.25)	1.00 (0.00, 2.00)	< 0.001
Oxford knee score (0–48)	29.7 ± 2.0	38.1 ± 2.7	< 0.001	29.1 ± 2.5	39.9 ± 2.2	< 0.001

**TABLE 4:** Postoperative variables comparisons.

Postoperation variables	“Two‐finger” method group (*n* = 26)	Measured resection group (*n* = 26)	*p*
Femoral angle A (°)	−3.38 ± 3.93	−1.79 ± 4.35	0.172
Tibial angle E (°)	−2.57 ± 3.12	−1.34 ± 2.57	0.218
Femoral angle B (°)	6.29 ± 2.33	6.44 ± 2.82	0.770
Tibial angle F (°)	4.21 ± 2.81	4.80 ± 2.42	0.147
Range of motion (°)	120 (115,130)	130 (120,130)	0.007*
Range of motion improvement (°)	12.50 (8.75, 20.00)	15.00 (8.75, 2625)	0.429
Visual analog scale (0–10)	2.00 (1.00, 3.00)	1.00 (0.00, 2.00)	0.013*
Oxford knee score (0–48)	38.1 ± 2.7	39.9 ± 2.2	0.013*
Global perceived effect (1–7)	1.5 (1.0, 2.0)	1.5 (1.0, 2.0)	0.895

* *p* < 0.05.

## 4 Discussion

### 4.1 Major Findings of the Study

In this research, we excogitated a “measured resection” method for gap balancing in MB‐UKA. Our findings demonstrated that the measured resection method turned to be a feasible and reliable method to help achieving proper gap balance in MB‐UKA. Patients operated with measured resection method showed better ROM, VAS, and OKS scores at the 6‐month follow‐up than the traditional “two‐finger” method, achieving better knee joint function and pain relief. Furthermore, we had also noted some issues worthy of discussion during the application of the measured resection method.

Proper gap balance has long been a challenge for minimal invasive MB‐UKA [29]. The traditional “two‐finger” method is a generally used method to achieve gap balance in MB‐UKA and is mainly based on the experience of the surgeon. However, this method is difficult to replicate among different surgeons and may cause significant deviations [30]. Moreover, the friction coefficient of the surgical instruments can also affect the accuracy of the “two‐finger” method. Besides, if the patients presented with higher knee joint laxity, it will be difficult for the surgeon to achieve proper gap balance by using the same method, which will lead to higher risk of complications [11]. The novel “measured resection” method is the quantification of the traditional “two‐finger” method. It provides an auxiliary and quantitative way to choose meniscal bearing when the traditional method reaches an ambiguous conclusion (e.g., when 3‐ and 4‐mm bearings are both acceptable based on the perception of “two‐finger” method). This method is also more suitable for high joint laxity patients as it aims to restore normal anatomical structure rather than reaching a familiar hand feeling. For patients with varying degrees of knee joint laxity, the measured resection method adjusts the alignment of the knee joint and corrects varus deformity by adjusting the thickness of the femoral sizing spoon before tibial osteotomy. For instance, patients with higher joint laxity may require a thicker femoral sizing spoon (2 or 3 mm instead of 1 mm) during the assessment of ligament tension and joint laxity. This adjustment elevates the plane of tibial transverse osteotomy, thereby avoiding the need for thicker meniscal bearing and associated complications (such as periprosthetic fractures) that may arise from excessive osteotomy [31]. Once the appropriate femoral sizing spoon is determined, the patient's preosteotomy flexion gap is established, and the surgeon can proceed with the surgery according to the measured resection method.

As shown in Table 2, every bearing thickness difference between the measured resection method and the “two‐finger” method is less than 1 mm in all 26 knees, which means the principle of measured resection method does not contradict with the traditional method. Moreover, patients received measured resection showed better ROM, OKS, and VAS scores at 6‐month follow‐up than the traditional “two‐finger” method group. According to our experience, if there are significant differences between the two methods, an iatrogenic injury of anterior cruciate ligament (ACL) or MCL should be considered. The calculation results of the measured resection method can intuitively reflect the degree of intraoperative osteotomy. If the bearing thickness calculated by the measured resection method is less than 3 mm, insufficient osteotomy should be considered and may cause iatrogenic ligament damage by excessively pulling the knee joint ligaments when attempting to insert a 3‐mm feeler gauge. If the bearing thickness by the measured resection method is more than 7 mm, then there may be excessive osteotomy, which can lead to meniscal bearing dislocation or even failure of the surgery because even the thickest bearing might not be able to restore soft tissue tension.

### 4.2 Issues in Measured Resection Method Application

We also noticed some issues during the application of measured resection method. Noninteger values often appear in the calculation results by the measured resection method, but the thickness of meniscal bearings is in a continuous integer, which is necessary to analyze the digits after the decimal point. It is now generally accepted that a thinner pad is more appropriate without increasing the risk of dislocation, as it reduces tension on the MCL and avoids the transfer of pressure to the lateral compartment, which can lead to surgical failure [32, 33]. We preferred a thinner bearing when the number after decimal point is less than eight, while a thicker bearing was chosen when the number was greater than or equal to eight.

Notably, Patients 6 and 19 in Table 2 yielded identical calculation results, yet they ultimately required different thicknesses of meniscal bearings. During Patient 6's surgery, a 3‐mm trial meniscal bearing was inserted based on the measured resection method, but it turned out to be too relaxed and might increase the risk of dislocation. A thicker 4‐mm trial meniscal bearing was appropriate and stable. This was verified by the traditional two‐finger method that a 4‐mm feeler gauge would be suitable. This result had drawn our attention to the calculation result with a decimal point of 7. As we can see, Patients 1, 4, 8, 13, 19, and 22, whose measured resection calculated results ended in a decimal of 7, yielded two potential bearing thicknesses through traditional two‐finger method assessment—either thicker or thinner. This suggests that a decimal of 7 in the measurement result might be an ambiguous point. While we tend to use thinner bearings to mitigate the risk of OA in the contralateral compartment, we cannot afford to ignore the significantly increased risk of bearing dislocation by refraining from using thicker bearings. It would be great if we could adopt more precise bearings to solve this problem, such as 3.5‐mm bearings or customized ones. We still need a larger sample size to adjust the decimal point threshold of measured resection method.

We can observe that the follow‐up OKS between the two patient groups exhibits a small numerical difference (38.1 vs. 39.9), yet this difference is statistically significant (*p* < 0.05). This may be related to the sample size, data variability, and statistical power of this study. Based on sample size calculations, 52 patients were recruited in this study, representing an appropriate number. The postoperative OKS scores (mean ± SD) for the two groups were 38.1 ± 2.7 and 39.9 ± 2.2, respectively, with small standard deviations indicating low dispersion among the data, which aids in more precise detection of subtle differences. The statistical power for the OKS scores, calculated using G*POWER, was 0.81, which is acceptable and allows for more reliable detection of small differences. However, these statistical characteristics are specific to this study, and further statistical analysis and elaboration will be necessary as more samples are included in subsequent studies. In this study, an OKS difference of 1.8 points demonstrated statistical significance and also showed some clinical superiority. On average, patients in Group 2 ranked 1–2 levels higher on 1–2 items of the OKS questionnaire, reflecting less knee pain or better functional status. For instance, if a patient can walk pain‐free for a longer duration and experiences less daily knee pain compared to others, their reported postoperative satisfaction will also be correspondingly higher. Therefore, despite being small, the difference in OKS scores holds promising clinical significance. Of course, we anticipate further improvements in patients' OKS scores in future studies, enabling them to better benefit from MB‐UKA.

### 4.3 Prospects and Limitations

Given the errors associated with traditional method for soft tissue balancing in the past, as well as the risks of postoperative complications and surgical failure, the measured resection method introduced in this study demonstrates superior postoperative outcomes and holds significant clinical importance. It optimizes the soft tissue balancing process in MB‐UKA, positively impacting patients' short‐term rehabilitation and quality of life, with potential long‐term benefits and broad application future. If the measured resection method is further proven successful in future research, it will profoundly influence the clinical practice of MB‐UKA. Due to its simplicity, ease of implementation, and requirement for no special instruments, the measured resection method can be widely adopted in clinical practice. For experienced surgeons, this new method may serve as a beneficial supplement, enhancing their ability to handle complex cases with greater ease. For novice surgeons, it may be a more accessible tool, as it simplifies surgical steps, improves surgical success rates, and potentially reduces the learning curve. For patients, both routine cases and those with complex conditions such as high joint laxity can fully benefit, extending the advantages to a broader population of patients with AMOA. Furthermore, the measured resection method has the potential to become a standard process of MB‐UKA procedures, enabling precise and quantifiable soft tissue balancing, thus resulting in better clinical outcomes.

The measured resection method also has some limitations in actual use. If non‐standard AMOA situations such as excessive osteophytes in the posterior femur are encountered, the measured resection method will be affected, the surgeons might fail to accurately measure the resected bones. Besides, if multiple osteotomies are performed on the tibial plateau and posterior condyle of the femur, resulting in the inability to obtain a complete bone specimen, the measured resection method would also be inapplicable. This requires the surgeon to perform precise osteotomy and demand certain experience. Furthermore, ACL incompetence combined with cartilage damage in the posterior tibia may also come across, and preoperative MRI and X‐ray imaging assessment or intraoperative compensation by reconstruction of ACL or convert into TKA are needed [34, 35].

The limitations of this study are as follows: first, the postoperative follow‐up time of this experiment is relatively short. Although statistically significant differences have been observed in the 6‐month follow‐up between the two groups of patients, the current findings can only indicate that patients using the measured resection method can achieve better short‐term therapeutic effects. Further follow‐up is required to evaluate multiple indicators such as long‐term functional status, revision rate, and complications. Second, the sample size of this study was relatively small. The study only included a partial population from a single center, and factors such as different racial groups, various prosthesis manufacturers, the use of cementless prostheses, were not adequately explored. Besides, to ensure consistency in other surgical factors, all operations in this study were performed by a single experienced surgeon. However, the study did not investigate whether surgeons with varying levels of experience could achieve comparable and satisfactory surgical outcomes using the measured resection method, the findings may not be universally applicable to all groups of surgeons. A larger sample size is needed to better explore, evaluate, and refine the measured resection method, making its results more convincing.

In conclusion, this study can serve as a pilot study to verify the feasibility of the new measured resection method in MB‐UKA. Further work will be needed to clarify whether this new method is beneficial for our patients and doctors.

## 5 Conclusion

The measured resection method preliminarily turned out to be an easy and reliable way to assist surgeons in choosing ideal meniscal bearing thickness in MB‐UKA to achieve proper gap balance and gain better clinical outcomes. As research continues, the measured resection method has the potential to become a reliable, easy‐to‐operate, and standardized soft tissue balancing method in MB‐UKA that can be widely used in clinical practice to benefit the patients.

## Author Contributions

All authors had full access to the data in the study and take responsibility for the integrity of the data and the accuracy of the data analysis. Q.L., J.R., W.Z., and R.H.: conceptualization. K.W. and R.H.: methodology. Q.L., J.R., W.Z., Y.L., Z.W., and J.H.: investigation. Q.L., W.Z., and S.X.: formal analysis. Q.L. and J.R.: writing – original draft. Q.L. and S.X.: visualization. K.W., T.L., and R.H.: supervision. K.W. and R.H.: funding acquisition. All authors read and approved the final manuscript.

## Ethics Statement

The methods and procedures for our study were approved by the Ethics Review Committee of the Third Affiliated Hospital of Sun Yae‐Sen University, study number II 2024‐011‐01. All participants included signed informed consent regarding publishing their data and photographs. This study was registered on ClinicalTrials.gov and all procedures were performed in accordance with the World Medical Association Declaration of Helsinki.

## Conflicts of Interest

The authors declare no conflicts of interest.
